# Asynchronous local differences in Lyme disease environmental hazard driven by variation in tick abundance and *Borrelia burgdorferi* infection prevalence

**DOI:** 10.1186/s13071-026-07509-8

**Published:** 2026-07-15

**Authors:** Jessica L. Hall, Agata Delnicka, Kevin J. Bown, Richard J. Birtles

**Affiliations:** 1 Human and Natural Sciences, School of Science, Engineering and Environment, University of Salford, Salford, M5 4WT UK; 2 Disease Control, Moredun Research Institute, Pentlands Science Park, Bush Loan, Penicuik, Midlothian EH26 0P UK; 3 Biological and Environmental Sciences, Liverpool John Moores University, Liverpool, L3 5AH UK

**Keywords:** Tick-borne disease, Lyme borreliosis, *Ixodes ricinus*, *Borrelia burgdorferi*

## Abstract

**Background:**

The tick-borne disease Lyme borreliosis (LB) is a clear and increasing public health threat across the temperate regions of the northern hemisphere, including the United Kingdom. The epidemiology of LB is shaped by the environmental hazard posed by its causative agent *Borrelia burgdorferi* sensu lato, which is frequently quantified in terms of the density of infected nymphal ticks (DIN).

**Methods:**

We report quantification of the dynamics of DIN at three sites in close proximity to one another in Cumbria, a region of the UK where LB is frequently reported, through monthly surveys of questing *Ixodes ricinus* nymphs over a 33-month period.

**Results:**

Despite the proximity of study sites, we observed significant spatial variation in DIN between them. We also observed significant temporal variation in DIN at all three sites, but this variation was asynchronous. Changes in both tick abundance and *B. burgdorferi* infection prevalence underlay this variation.

**Conclusions:**

These observations suggest very local key ecological determinants of DIN and that meaningful estimation/prediction of LB risk on a national or regional scale is extremely challenging.

**Graphical Abstract:**

**Figure:**
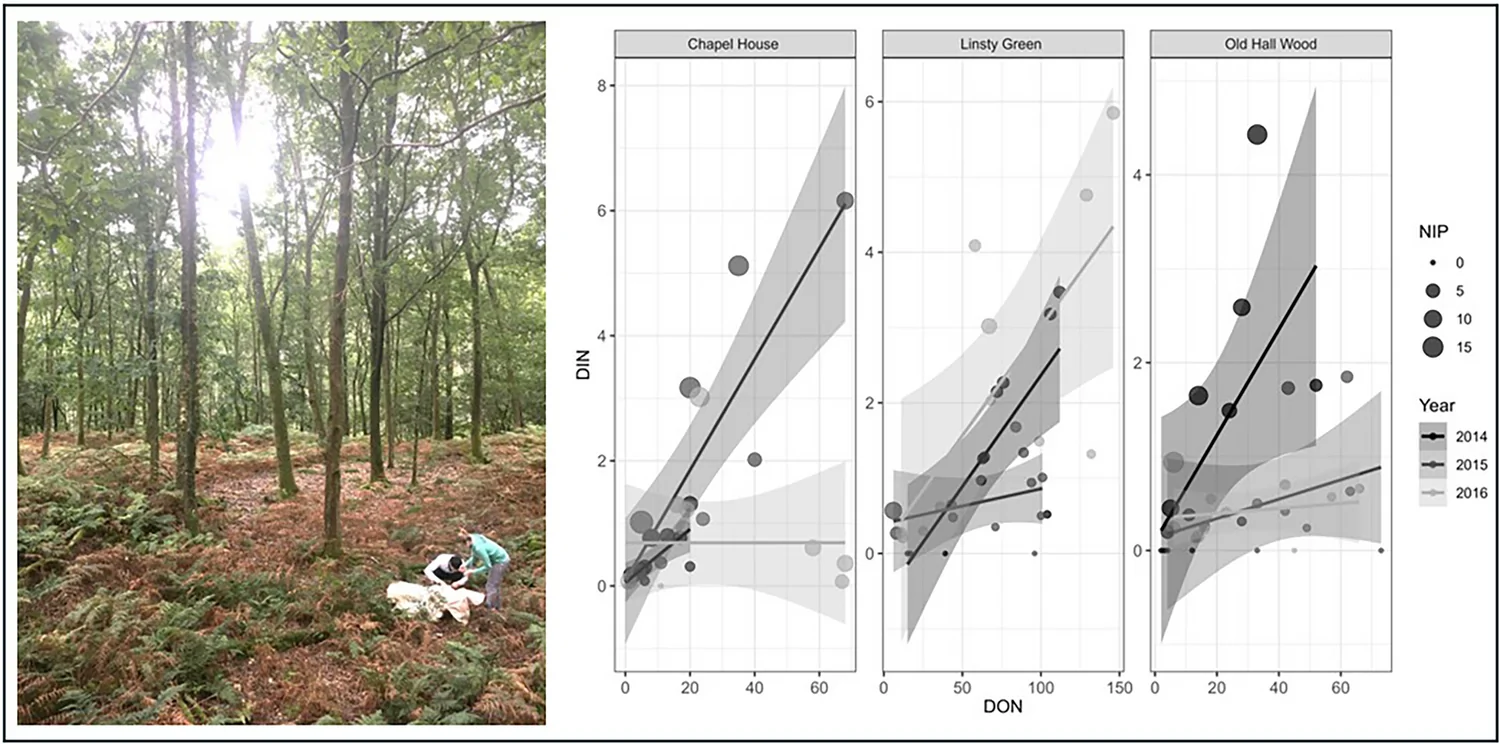

**Supplementary Information:**

The online version contains supplementary material available at https://doi.org/10.1186/s13071-026-07509-8.

## Background

Lyme borreliosis (LB), caused by the bacterium *Borrelia burgdorferi* sensu lato (s.l.) and transmitted by Ixodid ticks, is a zoonosis of established and rising public health concern in temperate regions of the northern hemisphere [1]. The incidence rate of LB in Europe is estimated to be 56.3 per 100,000 persons, but with significant spatial variation on a continental scale and within constituent countries [1–9]. This variation reflects the frequency with which humans encounter ticks in particular locations, and the abundance of ticks that are infected with *B. burgdorferi* s.l.; this second parameter can be defined as “environmental hazard” and is quantified in terms of the density of infected nymphs (DIN) at a site [10]. As consequence of this, the literature reports on the density of nymphs (DON), the infection prevalence of *B. burgdorferi* s.l. in nymphal ticks (NIP) and the resulting DIN.

Quantification of DON at sites around the UK has demonstrated marked spatial variation, with surveys revealing between 0 and 16 nymphs per 100m^2^ [10–17]. NIP in questing *I. ricinus* populations has also been surveyed at numerous sites around the UK and, as with DON, marked variation has been reported. In some locations, no evidence of *B. burgdorferi* infections could be found in ticks, even if ticks were abundant, whereas elsewhere as many as 24% of ticks were found to be infected with the mean infection prevalence in the UK around 5.1% [13–25]. Far fewer studies have combined DON and NIP to estimate DIN, but those that have reported a range of between 0 and 12 infected nymphs per 100m^2^ at sites across the country [17, 21, 23].

Systematic quantification of spatial variation in DIN at sites across the UK could ultimately generate national risk maps of Lyme disease environmental hazard but represents a huge undertaking. An alternative approach to creating such maps is to identify the determinants that underlie DIN and then use this information to predict hazard. This approach is also challenging due to the complexity of *B. burgdorferi* ecology [26], particularly in Europe where multiple genospecies of *B. burgdorferi* s.l. adapted to different niches, circulate [27].

Of fundamental importance to unpacking *B. burgdorferi* environmental hazard is to understand if variation in DIN is primarily driven by variation in DON or variation in NIP, or both. If, for example, DON drives DIN, then interventions aimed at reducing tick abundance should reduce this hazard, but if DIN is not significantly influenced by DON, investment in such interventions would be ineffective. Various studies have addressed this issue by concurrent survey of multiple sites, and there does appear to be an overall relationship between DIN with DON [28, 29]. However, exceptions were not uncommon, with unexpectedly high or low DIN when DON was considered [25, 30, 31]. Correlation between DON and NIP appears to be weaker [25, 28, 31, 32].

An additional route to identifying the ecological determinants of DIN is to consider the spatial and temporal scales on which DIN variation occurs. If DIN is constant over a large area, this suggests a response to a wide-acting determinant such as rainfall, whereas if DIN dynamics are localised this suggests key determinants vary significantly over short distances such as small mammal abundance. To date, explicit exploration of the scale on which spatial variation in DIN occurs has been limited beyond demonstration of significant differences at a national or regional (state/county) level [15, 28, 31]. However, a few studies have quantified variation in DIN on a more local scale, for example, in/around a particular town/city [33] or across a geographically restricted landscape [25]. Even finer scale variation in DIN has been observed between transects separated by a few hundred metres [32, 34, 35]. Assessing the temporal stability of DIN is important not only for informing ecological models but also for assessing the value of using data collected for one year to accurately represent the longer-term environmental hazard posed by a site. If DIN at a site varies dramatically from year to year, the value/accuracy of empirically generated risk maps will be compromised.

Longitudinal studies have catalogued temporal variation in DIN both within a single season [31] and over multiple consecutive years [e.g. 25, 28, 29, 31]. Although these latter studies observed some degree of consistency overall, this trend was interrupted by exception years. Furthermore, the overall consistency of DIN often masked marked asynchrony between sites and juxtaposed concurrent changes in DON and NIP.

In order to contribute to the body of evidence summarised above, this study aimed to measure DON, NIP and DIN dynamics over a 33-month period at three sites that lay in close proximity to one another in north-western England to explore the relative importance of DON and NIP in shaping DIN dynamics.

## Methods

### Study sites

**Fig. 1:**
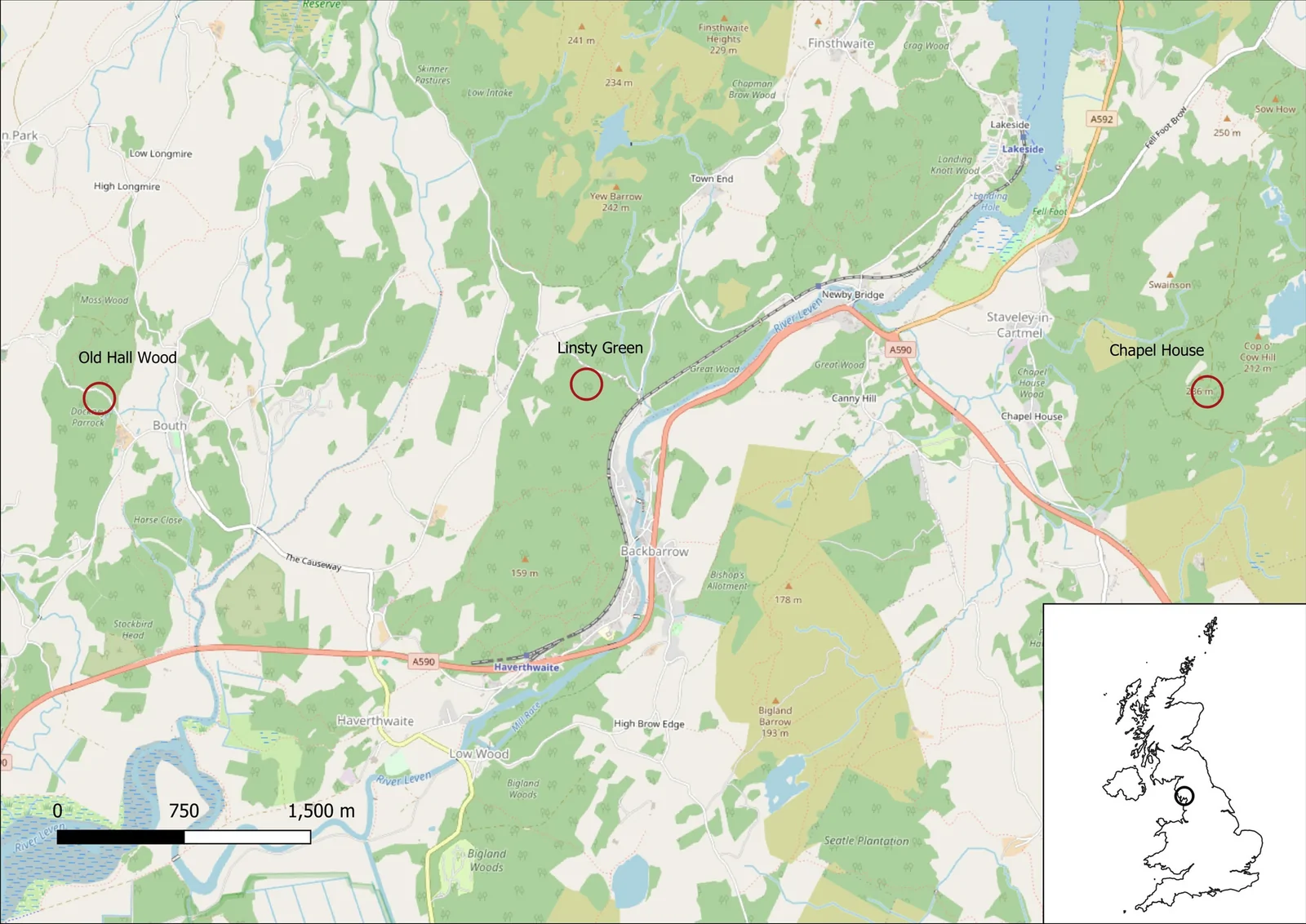
Location of the three field sites used in the study. The Figure was generated using QGIS 3.42 “Münster” [57]

Three sites were surveyed in southern Cumbria (Fig. 1). Sites were selected based on their proximity to one another, varying deer populations (determined by numbers culled at each site for deer management purposes), habitat type and the presence of ticks on initial survey. Chapel House (site 1) 54°15′53.6"N 2°56′31.2"W was an upland pine forest/ grassland area (elevation 200 m). Old Hall Wood (site 2) 54°15′45.8"N 3°02′20.9"W was a lowland, primarily deciduous woodland (elevation 90 m). Linsty Green (site 3) 54°15′55.2"N 2°59′33.6"W was a lowland mixed woodland (elevation 70 m). All three sites are within a 7 km distance from one another (Fig. 1). Forestry England categorised these sites as low (Chapel House), medium (Old Hall Wood) and high (Linsty Green) for deer numbers based on their estimates of population sizes in 2013.

### Sample collection

Questing *I. ricinus* nymphs were collected approximately every 4 weeks on rain/snow-free days between January 2014 and November 2016. Ticks were collected using a simple drag technique [36]. A woollen blanket of 1.6 m width × 2.0 m length was dragged across the woodland floor for 10 m, then turned over and examined for ticks. All adult and nymphal ticks observed on the blanket were removed into 70% ethanol. This process was repeated nine times at different parts of the site after which the total number of nymphs and adults collected was recorded to get a total number of ticks across 320m^2^ area of each site. If less than 200 nymphs were collected during these first 10 drags, further drags were completed until 200 nymphs were obtained or until 60 min had been spent at the site. The time and air temperature were recorded at each site once dragging had been completed and a brief description of the weather and ground conditions made.

### DNA extraction and Borrelia burgdorferi s.l. PCR

DNA extraction was performed in a dedicated laboratory located apart from those in which PCR products were handled. Each *I. ricinus* nymph was placed in an individual 1.7 ml microcentrifuge tube, and a crude DNA extract was prepared from it using alkaline lysis as previously described [37]. One cross contamination control (no tick) sample was co-processed with every four ticks. Extracts were individually tested for the presence of *B. burgdorferi* s.l. species by real-time PCR amplification of a fragment of the 23S ribosomal RNA encoding gene, as previously described [38]. All assays included both negative (no DNA) and positive controls.

### Statistical analysis

All statistical analysis was performed in the software R version 4.4.0. Generalized linear models (GLMs) of DON and DIN were carried out using the package MASS and the GLM of NIP was carried out in the package glmmTMB, due to its ability to handle betabinomial distributions.

Season, rather than month, was added to all models as a factor, so as not to overfit the model whilst accounting for the seasonal variation present in tick numbers and prevalence. Each month within each site and year, was only sampled once. As such each season per site and year only contains 3 observations, or 2 if a given month was not sampled due to inclement weather conditions. Winter was classed as months between December and February, Spring as months between March and May, Summer as months between June and August, and Autumn as months between September and November. The first negative binomial (log link) GLM explored the relationship between DON and the effects of season, site and year. An interaction was used between site and year to understand if DON at each site depends on year. The second negative binomial (log link) GLM explored the relationship between DIN and the same fixed effects as the first. The NIP model used a betabinomial distribution (log link) explored the relationship between NIP and the same fixed effects as the other models. Model residuals were checked using the DHARMa package, and for the NIP model a betabinomial distribution was chosen due to a better fit than a binomial distribution. Pairwise comparisons using Tukey p-value adjustment of estimated marginal means within each site were calculated using the emmeans and pairs functions from the package emmeans. Prevalence and 95% confidence intervals for the prevalence were calculated using the Clopper and Pearson method using the epi.conf function from the epiR package.

At some of the sampling points in Chapel House, insufficient numbers of ticks (less than 2 ticks) were tested for the presence of *B. burgdorferi* s.l. This meant that NIP could not be estimated for certain months (January 2014, February 2014, December 2015, January 2015, December 2016), and, therefore, DIN could not be estimated in December 2016 (DON > 0 ticks) but was estimated as 0 for other months (DON = 0 in each of January 2014, February 2014, December 2015, January 2015). Where appropriate, these values are included in the figures displaying longitudinal patterns but excluded from NIP and DIN estimates and statistical analyses.

## Results

**Fig. 2:**
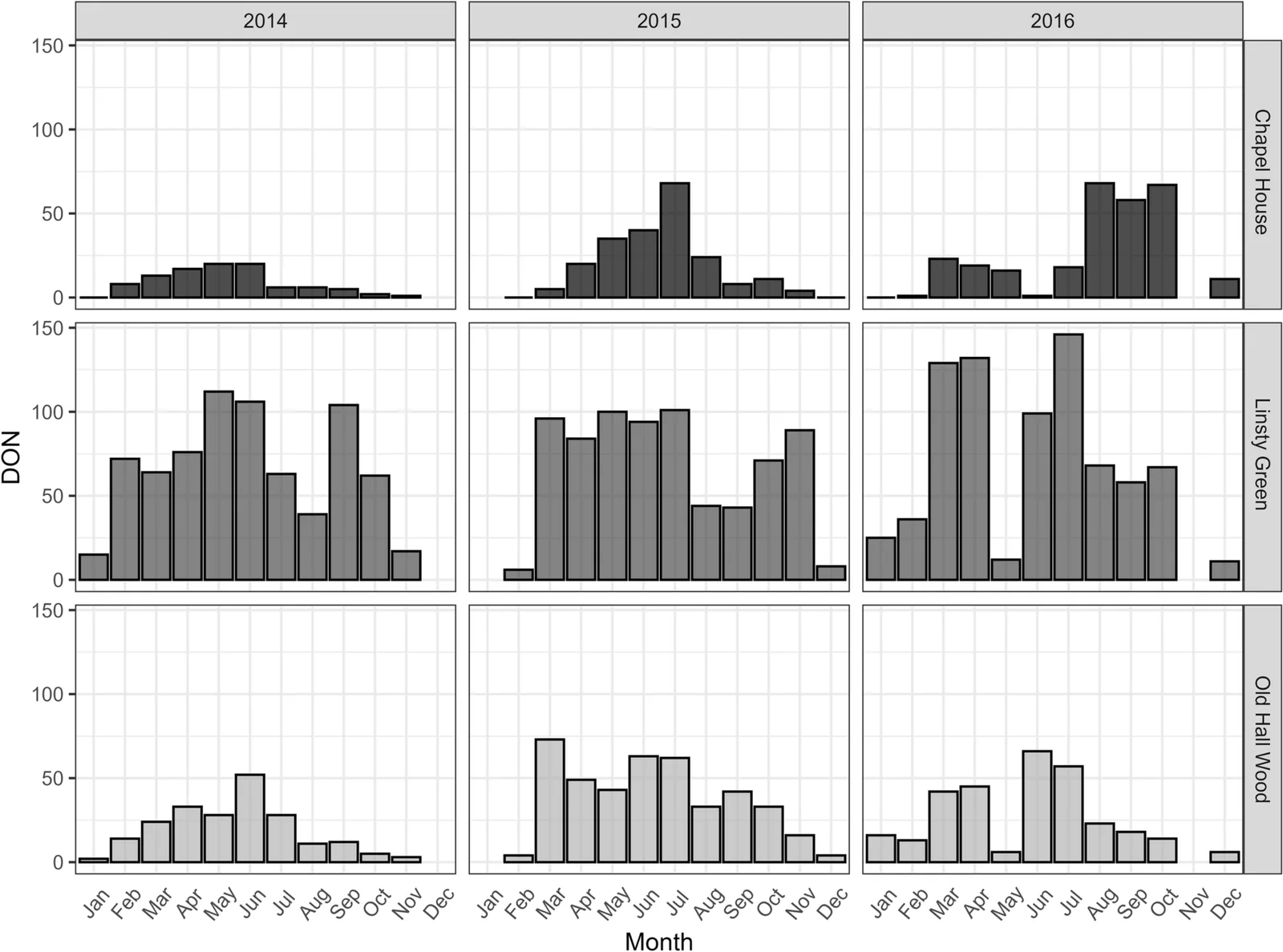
Observed DON at Chapel House, Linsty Green and Old Hall Wood from January 2014 to December 2016 at four-week intervals

Between January 2014 and December 2016, 33 surveys were conducted on each of the three study sites. Occasional planned surveys were abandoned due to poor weather (December 2014 and January 2015) (Fig. 2). In total 12,381 nymphs and 1235 adults were collected (larvae were not quantified). A total of 2687 nymphs and 149 adults were collected at Chapel House, 4233 nymphs and 216 adults from Old Hall Wood and 5461 nymphs and 870 adults from Linsty Green. Thus, the overall ratio of nymphs to adults was 10.4:1 but this ratio varied markedly between sites; 18.6:1 at Chapel House, 20.0:1 at Old Hall Wood and only 6.6:1 at Linsty Green.

### Density of questing nymphs (DON)

**Fig. 3:**
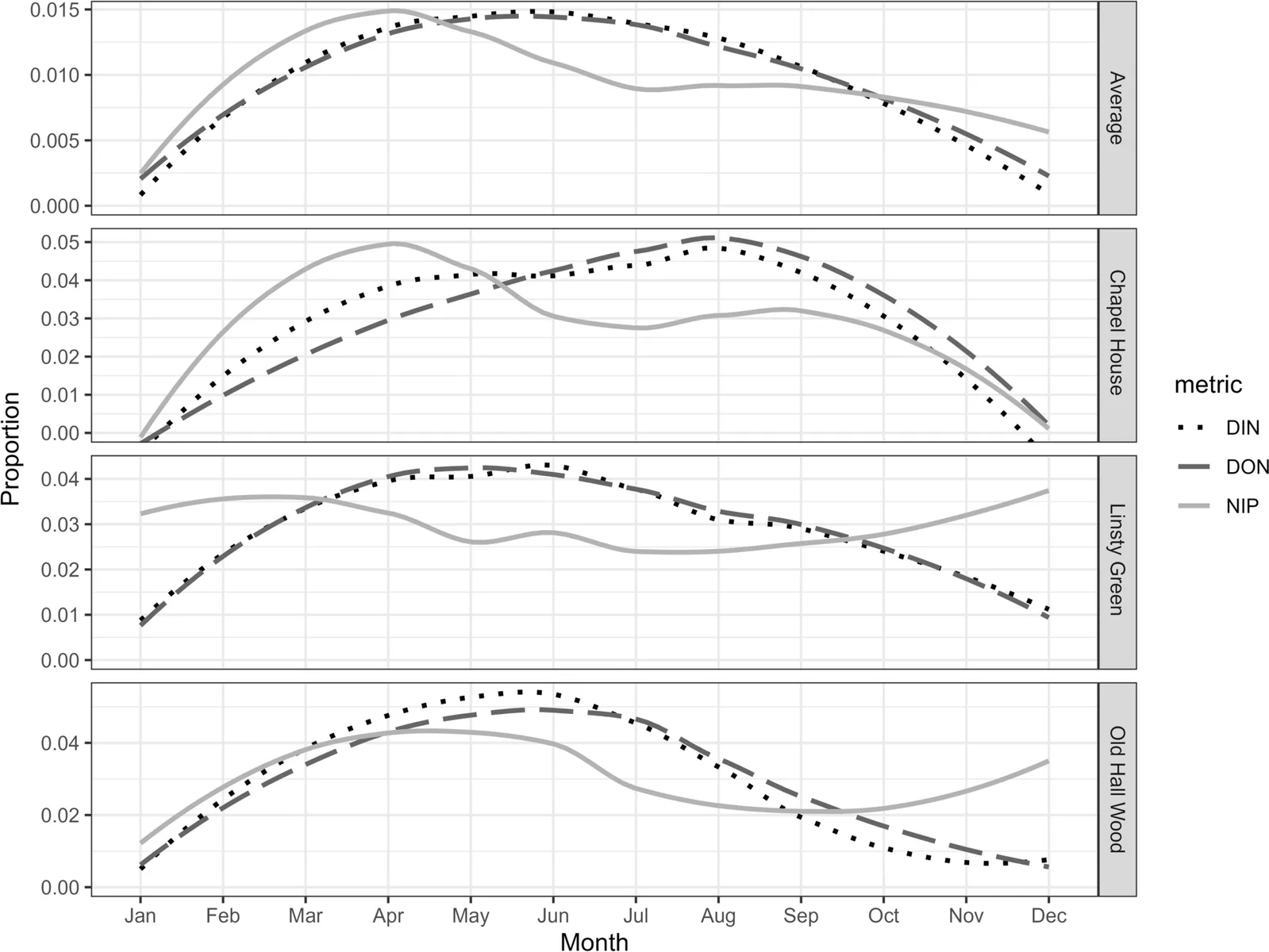
A combined phenology for each site for all years and an “average” graph representing all sites for all years, with the relative proportion of each metric (DIN, DON, NIP) for any given month per each site

Questing ticks were encountered throughout the year but were significantly less abundant in winter months (November to January) (Table 1). Typically, DON started to rise in February, peaking between March and July then gradually declining towards November (Figs. 2 and 3). Overall, the nymphal phenologies at all 3 sites appeared reasonably synchronous, although that at Chapel House was somewhat delayed (Fig. 3). Nymphal density typically peaked in July, although atypical patterns were observed; for example, at Chapel House in 2016, nymphs were far more abundant in late summer/early autumn than earlier in the year. A combined phenology for all sites/years appeared unimodal (Fig. 3), although a short-lived but marked drop in DON was sometimes observed in mid-summer, for example at all sites in 2016 (Fig. 2).

**Table 1:** Parameter estimates and standard errors for the negative binomial model of DON

Parameter	Estimate (SE)	*z* value	*p* value
Intercept	0.892 (0.274)	3.253	0.001
Spring	1.535 (0.206)	7.433	< 0.001
Summer	1.662 (0.206)	8.063	< 0.001
Autumn	1.055 (0.215)	4.919	< 0.001
Linsty Green	2.196 (0.3)	7.319	< 0.001
Old Hall Wood	0.765 (0.308)	2.488	0.013
2015	0.6 (0.309)	1.941	0.052
2016	1.159 (0.306)	3.787	< 0.001
Linsty Green *2015	− 0.828 (0.418)	− 1.980	0.048
Old Hall Wood*2015	0.017 (0.425)	0.041	0.967
Linsty Green *2016	− 1.193 (0.415)	− 2.874	0.004
Old Hall Wood*2016	− 0.688 (0.424)	− 1.623	0.105

Baselines used were 2014, Winter and Chapel House. The ‘*’ indicates an interaction term between given parameters

Overall mean annual DON for the study was 38.22 nymphs per 100m^2^ ± 3.55 (SE). However, significant differences between sites were evident, with all pairwise comparisons between sites being significant, Chapel House had a lower DON than Old Hall Wood (ratio = 0.58, SE = 0.1, *z* = − 3.15, *p* = 0.005 and Linsty Green (ratio = 0.22, SE = 0.04, *z* = − 9, *p* < 0.001). Linsty Green had higher DON than Old Hall Wood (ratio = 2.67, SE = 0.44, *z* = 5.92, *p* < 0.001) (Table 1). Across the sampling period, the mean DON was 18.00 nymphs per 100m^2^ ± 3.56 (SE) for Chapel House, 28.50 nymphs per 100m^2^ ± 3.67 (SE) for Old Hall Wood, and 68.20 nymphs per 100m^2^ ± 6.76 (SE) for Linsty Green. Mean DON for each site and year is shown in Additional file 1: Table S1.

A significant statistical interaction was present between site and year, implying that year affects DON at sites differently. Specifically, at Chapel House DON peaked in 2016 (25.64 (SE = 7.89)) and was almost three times higher than that in 2014 (8.91 (SE = 2.24)**;** *z* = − 3.79, *p* < 0.001), with no statistical differences in DON at Chapel House between 2014 and 2015 (ratio = 0.55, SE = 0.17, *z* = − 1.94, *p* = 0.13), or 2015 and 2016 (ratio = 0.57, SE = 0.17, *z* = − 1.88, *p* = 0.14). Despite fluctuations of DON across years at Old Hall Wood and Linsty Green, there were no statistical differences in DON across years within those sites.

DON was significantly affected by season, with both spring and summer DON being higher than each of winter and autumn (Table 1, Fig. 3).

### Nymphal infection prevalence (NIP)

A total of 428 of the 12,381 nymphs tested were found to contain *B. burgdorferi* s.l. DNA and were thus considered infected (3.45%, 95% confidence intervals (CIs) = 3.14–3.79%).

**Fig. 4:**
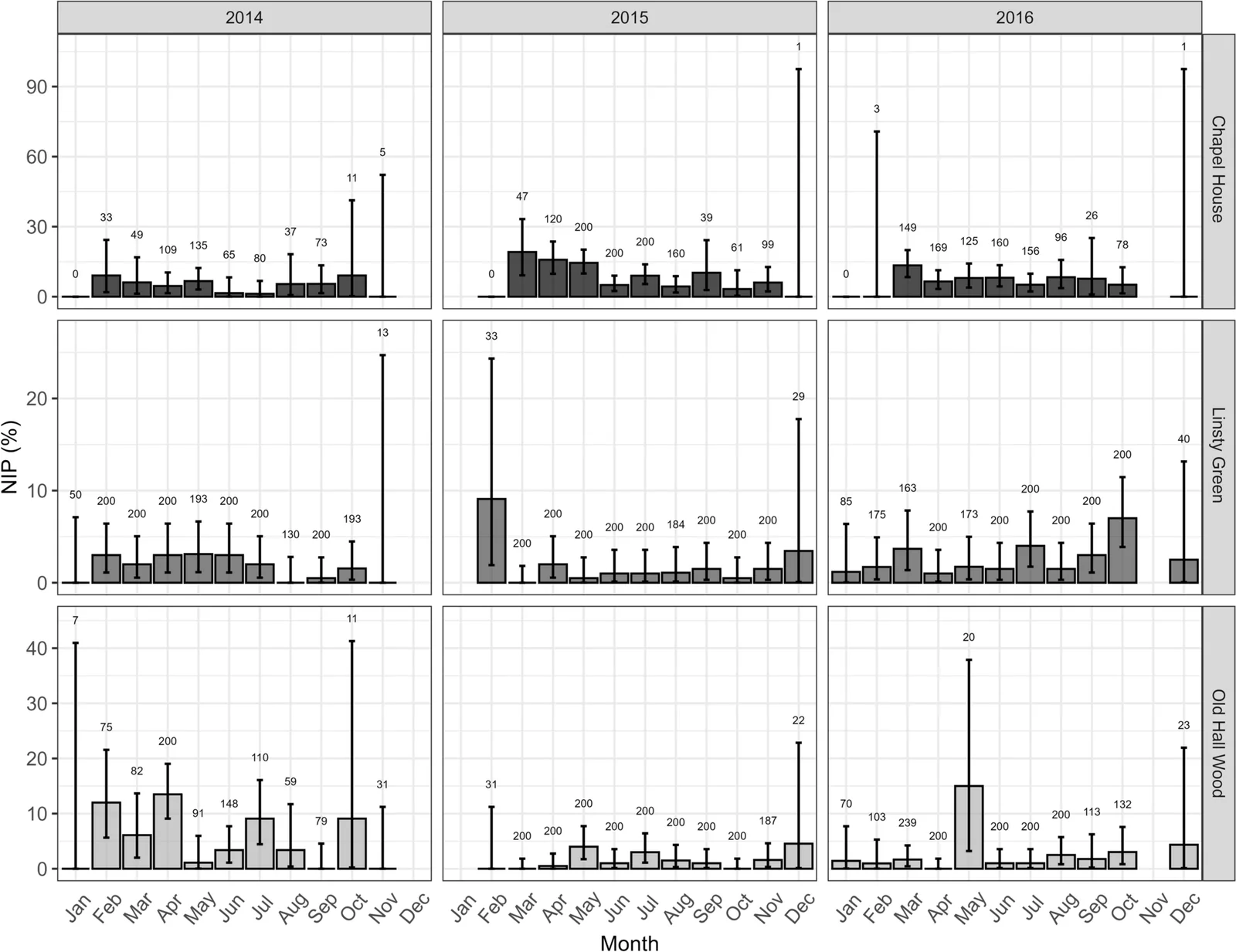
Observed NIP calculated at Chapel House, Linsty Green and Old Hall Wood from January 2014 to November 2016 at four-week intervals. The number over each bar represents how many nymphs were tested at that time point

There were seasonal fluctuations in overall NIP (data from all three sites combined, Fig. 3), demonstrating a significantly higher NIP in spring than in summer (OR = 1.48, SE = 0.22, *z* = 2.62, *p* = 0.04) but not across any other pairwise comparisons (Table 2). However, a trend was apparent, with NIP rising through the spring/early summer, then declining before a brief mid-autumn resurgence. This trend was apparent at the site level for Chapel House, but less so at Old Hall Wood and Linsty Green (Fig. 3). At Chapel House, there was an observable pronounced peak in the spring (Figs. 3 and 4). However, this apparent trend was derived from relatively small monthly sample sizes and was far less pronounced when the average NIP for all sites was calculated, although a decline in the later months remained (Fig. 3).

**Table 2:** Parameter estimates and standard errors for the betabinomial model of NIP

Parameter	Estimate (SE)	*z* value	*p* value
Intercept	− 2.77 (0.304)	− 9.102	< 0.001
Spring	− 0.069 (0.242)	− 0.284	0.777
Summer	− 0.46 (0.25)	− 1.84	0.066
Autumn	− 0.476 (0.274)	− 1.739	0.082
2015	0.705 (0.254)	2.776	0.006
2016	0.503 (0.262)	1.92	0.055
Linsty Green	− 0.875 (0.309)	− 2.831	0.005
Old Hall Wood	0.194 (0.282)	0.689	0.491
2015*Linsty Green	− 1.066 (0.423)	− 2.52	0.012
2016*Linsty Green	− 0.271 (0.395)	− 0.686	0.492
2015*Old Hall Wood	− 2.122 (0.41)	− 5.171	< 0.001
2016*Old Hall Wood	− 1.684 (0.406)	− 4.152	< 0.001

Baselines used were 2014, winter, Chapel House. The ‘*’ indicates an interaction term between given parameters

NIP was highest at Chapel House where 209 nymphs of the 2687 tested were positive for *B. burgdorferi* s.l. (7.78% (95% CI = 6.79%–8.86%). NIP at Chapel House was 2.93-fold (SE = 0.49) higher than at Old Hall Wood (111 nymphs positive out of 4233 tested, NIP = 2.62% (2.16%, − 3.15%); *z* = 6.42, *p* < 0.001) and 3.74-fold (SE = 0.62) higher than that at Linsty Green (108 nymphs were positive out of 5461 tested, NIP = 1.98% (1.63–2.38%); *z* = 7.97, *p* < 0.001) (Table 2).

However, there was a significant interaction between site and year indicating that NIP peaked in different years at each site (Table 2). Annual fluctuations of NIP within site were apparent for Chapel House and Old Hall Wood. In Chapel House, NIP in 2014 was almost half that of 2015 (OR = 0.49, SE = 0.13, *z* = − 2.78, *p* = 0.02), but there were no statistically significant differences in NIP between 2014 and 2016 (OR = 0.6, SE = 0.16, *z* = − 1.92, *p* = 0.13), or 2015 and 2016 (OR = 1.22, SE = 0.24, *z* = 1.01, *p* = 0.57), indicating that the peak in NIP was similarly high in 2015 and 2016. In Old Hall Wood, NIP peaked in 2014 and was 4.12-fold higher (SE = 1.33) than that in 2015 (*z *= 4.4, *p* < 0.001) and 3.26-fold higher (SE = 1) than that in 2016 (*z* = 3.85, *p* < 0.001). Despite small fluctuations in NIP between years within Linsty Green, these differences were not significantly different.

### Density of infected nymphs (DIN)

The overall mean annual DIN for the study was 1.16 infected nymphs per 100m^2^ ± 0.15 (SE). Data from all three sites indicated that DIN showed a unimodal seasonal pattern rising in the spring to peak in early summer before decreasing markedly thereafter (Fig. 3). DIN in winter was significantly lower than that in spring (ratio = 0.23, SE = 0.09, *z* = − 3.57, *p* = 0.002) or summer (ratio = 0.24, SE = 0.1, *z* = − 3.43, *p* = 0.003). DIN in autumn was intermediate between the peak DIN in spring and summer and the low DIN of winter, with none of these differences being significantly different from each other (*p* > 0.05). Within summer, DIN in Chapel House appeared to peak later in the summer (August) than in Old Hall Wood and Linsty Green, where DIN appeared to be highest in early summer (June) (Fig. 3).

After correcting for multiple comparisons, DIN was not significantly different between sites, despite a marginal but non-significant trend of higher DIN at Linsty Green than in Old Hall Wood (ratio = 1.95, SE = 0.57, *z* = 2.29, *p* = 0.06) (Table 3). Chapel House had an annual mean DIN of 1.4 per 100m^2^ ± 0.32 (SE). Old Hall Wood had the lowest DIN with an annual mean of 0.72 per 100m^2^ ± 0.16 (SE). Linsty Green had the highest DIN with an annual mean of 1.37 per 100m^2^ ± 0.26 (SE).

**Table 3:** Parameter estimates and standard errors for the negative binomial model of DIN

Parameter	Estimate (SE)	*z* value	*p* value
Intercept	− 1.985 (0.595)	− 3.337	0.001
Spring	1.467 (0.411)	3.572	< 0.001
Summer	1.415 (0.412)	3.433	0.001
Autumn	0.922 (0.44)	2.095	0.036
Linsty Green	1.152 (0.544)	2.117	0.034
Old Hall Wood	0.998 (0.554)	1.802	0.072
2015	1.397 (0.531)	2.632	0.008
2016	1.522 (0.529)	2.875	0.004
Linsty Green *2015	− 2.059 (0.705)	− 2.921	0.003
Old Hall Wood*2015	− 2.206 (0.743)	− 2.968	0.003
Linsty Green *2016	− 1.087 (0.642)	− 1.693	0.091
Old Hall Wood*2016	− 2.479 (0.767)	− 3.232	0.001

Baselines used were 2014, winter, Chapel House. The ‘*’ indicates an interaction term between given parameters

As seen for DON and NIP, there was a significant interaction between site and year (Table 3), which highlighted the asynchronous interannual fluctuations in DIN within sites. Within Chapel House, DIN was lowest in 2014 relative to that in 2015 (ratio = 0.25, SE = 0.13, *z* = − 2.63, *p* = 0.02) and 2016 (ratio = 0.22, SE = 0.12, *z* = − 2.87, *p* = 0.01), but there was no significant difference in DIN between 2015 and 2016 (*z* = − 0.36, *p* = 0.93). In Old Hall Wood, none of the pairwise differences in DIN between years were significant, despite a trend for higher DIN in 2014 relative to 2015 (ratio = 2.25, SE = 1.17, *z* = 1.56, *p* = 0.27) and 2016 (ratio = 2.6, SE = 1.45, *z* = 1.72, *p* = 0.2). In contrast, at Linsty Green DIN was lowest in 2015 compared to 2016 (ratio = 0.33, SE = 0.15, *z* = − 2.49, *p* = 0.03), with intermediate DIN in 2014, which was not significantly different from DIN in 2015 (ratio = 1.94, SE = 0.9, *z* = 1.43, *p* = 0.33) or 2016 (ratio = 0.65, SE = 0.24, *z* = − 1.19, *p* = 0.46).

### Relative influence of DON and NIP on DIN

**Fig. 5:**
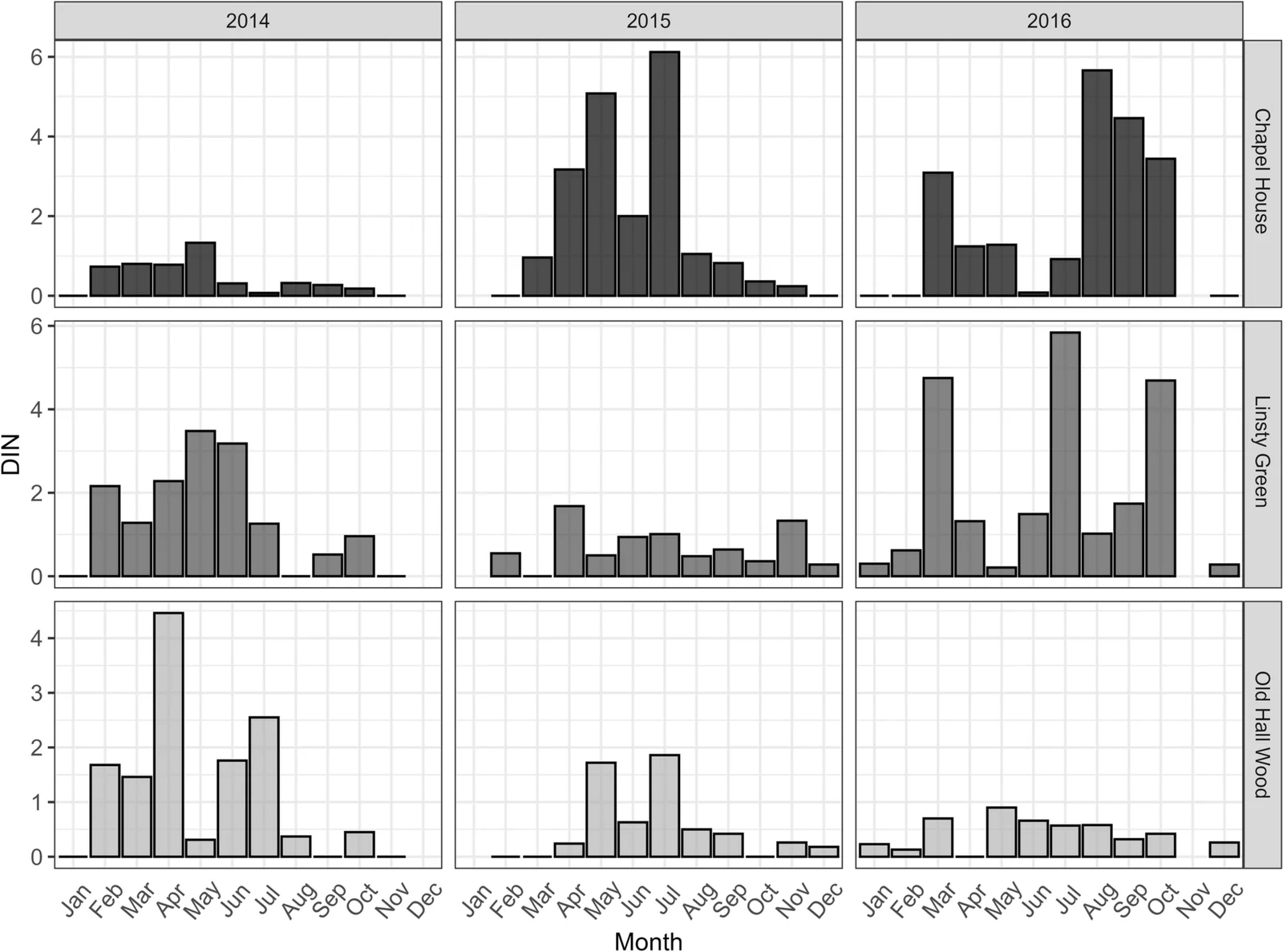
Observed DIN at Old Hall Wood, Chapel House, Linsty Green from January 2014 to November 2016 at four-week intervals

**Fig. 6:**
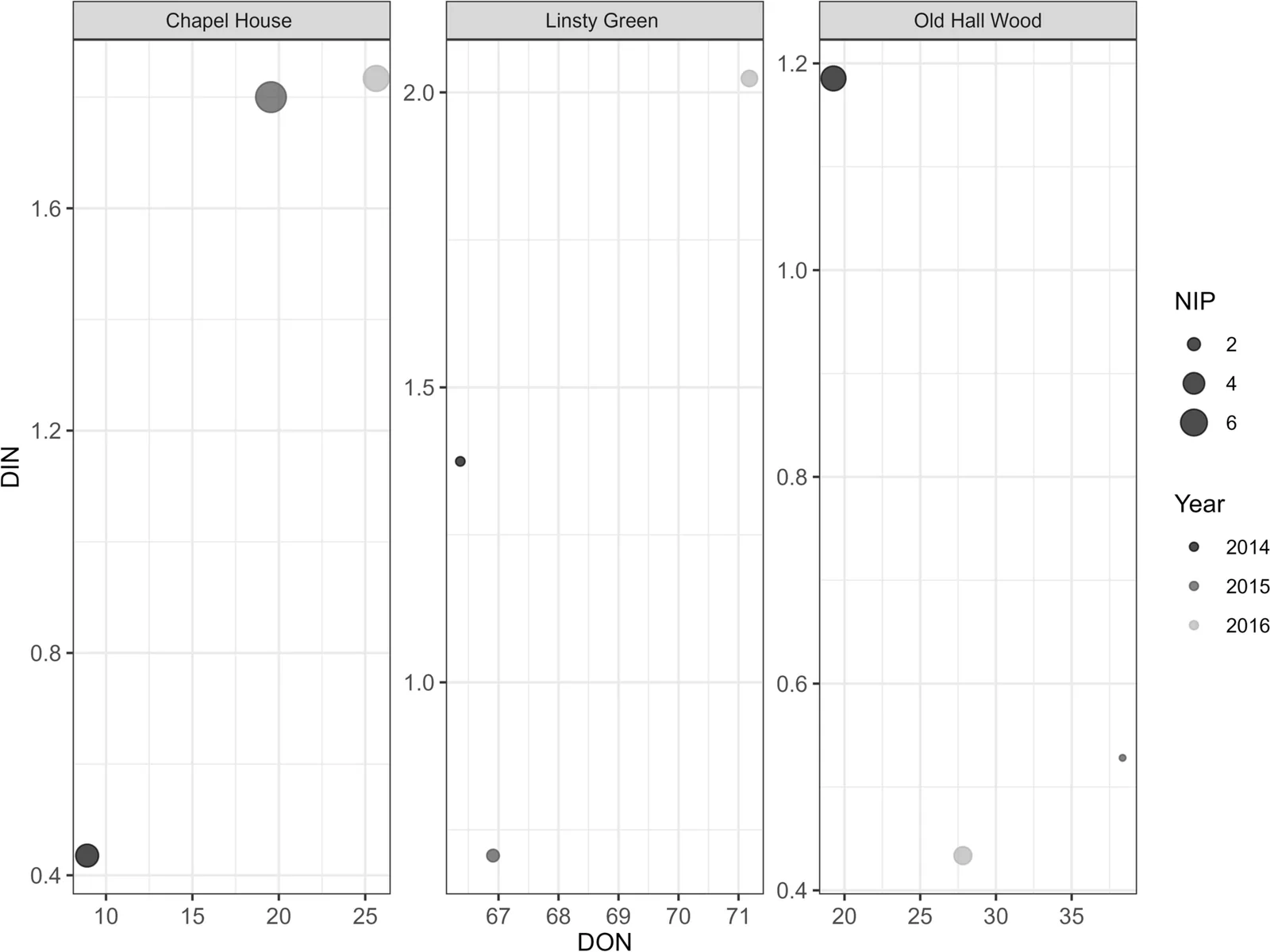
The relationships between observed DIN, DON and infection prevalence. Infection prevalence is indicated by the size of the circle, year by grey-scale colour

The results presented above indicate spatial and temporal variation in DIN across our study area. However, this differs by site (Figs. 5 and 6), with DIN at Chapel House being driven more by DON (Additional file 1: Fig. S1a) but NIP playing a larger role than DON at Old Hall Wood (Additional file 1: Fig. S1b). For example, at Old Hall Wood DIN in 2014 was twice that of other years despite DON being approximately half that observed in 2015 (Fig. 6). This peak therefore appeared more a consequence of a higher NIP year at the site. Conversely, the peak DIN years at Linsty Green (2016) and Chapel House (2015 and 2016), were associated with relatively high DON and relatively high NIP coinciding in those years (Fig. 6). Thus, peak DIN in two of our sites was primarily a consequence of DON and NIP being coincidently relatively high, yet the disproportionately larger contribution of NIP to peak DIN in Old Hall Wood in 2014 highlights that both DON and NIP are important determinants of DIN, despite their variable contributions to DIN across different sites and years.

## Discussion

This study quantified the dynamics of Lyme disease hazard by monthly surveys of questing *I. ricinus* ticks over a three-year period at three woodland sites in close proximity to one another in a region of north-west England where Lyme disease is endemic. The importance of longitudinal studies for exploring tick population dynamics, tick-borne pathogen ecology and the determinants of both, is well established [39]. Although the scale of the survey was relatively small, this limitation is offset by the generation of intensive datasets for each site that are strictly comparable because they were collected by the same person following an identical, prescribed field protocol; the value of this approach has been noted previously [10].

Questing *I. ricinus* nymphs were encountered on every one of the 33 survey days completed, albeit at near-zero abundances in some winter months. In total, 12,381 nymphs were collected from populations that had an overall annual mean density (DON) of 0.38 per m^2^. This DON value is within the range of those reported elsewhere in the UK [10, 13, 15, 21, 25, 40], although most previous estimates are inflated because they are derived from surveys carried out only during periods of high tick activity. The overall prevalence of *B. burgdorferi* infection in the nymphs collected (NIP) was 3.45%; again, this value is akin to those reported elsewhere in the UK [13, 15, 18, 19, 21, 25, 41]. Thus, for these key parameters at least, our sites do not appear abnormal and therefore the extrapolation of the data yielded from them is likely to be of value at a national level and beyond.

For our statistical analyses we focused modelling of the seasonal variation in DON, NIP and DIN by using a ‘season’ fixed effect in our GLMMs, rather than including ‘month’. This approach meant that we were unable to statistically compare monthly changes in DON, NIP and DIN. However, this approach was chosen to account for within-year variation within each site whilst ensuring ease of interpretation (i.e. comparing four seasons rather than 12 months) and thus avoiding overfitting of the model with the available data.

### Patterns across the study area

The pattern of seasonal activity by *I. ricinus* nymphs we observed broadly followed those previously described for England [10, 40] and elsewhere in Western Europe [e.g. 31, 42], with the density of questing nymphs (DON) rising through spring to a peak in early summer then gradually declining until the end of the year. The prevalence of *B. burgdorferi* infections in questing nymphs (NIP) appeared to fluctuate seasonally, with overall mean NIP rising through the spring to an April peak before gradually declining towards the end of the year. However, this decline appears to be interrupted by a modest ripple in September. Previous studies across Europe have noted both unimodal [31, 42] and bimodal [43] seasonal patterns for NIP. It has been proposed that these are a consequence of seasonal variation in (i) the abundance of *B. burgdorferi*-competent hosts, (ii) infection rates in these hosts, (iii) the impact of *B. burgdorferi* infections on tick activity, or (iv) the abundance of tick symbionts [31, 42–44]. The interaction of seasonal patterns of DON and NIP yielded a seasonal pattern for Lyme disease risk (density of infected nymphs, DIN). Given that overall mean NIP varies only minorly with season with notable differences present between spring and summer but not any other seasons, it is unsurprising that the seasonal pattern of overall mean DIN closely mirrors that of overall DON. This correlation has been observed elsewhere [31, 32, 42, 45], although not everywhere [43] and has been used to support the hypothesis that DON alone is a good indicator of Lyme disease hazard [45, 46]. However, caveats for this hypothesis have been raised [47].

We also explored inter-annual patterns of nymphal abundance, infection prevalence and Lyme disease risk in our study area. Patterns in DON at our sites varied by site, with notable year on year increases within Chapel House between 2014 and 2016, but this trend was less visible within other sites. A similar trend of increases in DON between 2014 and 2015, was reported for sites in southern England [25]. The authors suggested in this study that very heavy rainfall in January and February 2014 may have affected nymph survival [25] and the impact of severe weather on nymphal abundance has been proposed elsewhere [48]. However, rainfall in southern Cumbria during this period was close to normal [49]. Our results do, however, fit with an alternative suggestion by Medlock and colleagues [25] based on previous work correlating the scale of tree masting (high seed production) with ixodid tick abundance in North America and Europe [50, 51]. Medlock and colleagues [25] suggested that as 2012 was a low mast year, this resulted in a reduction in small mammal numbers and thus fewer tick larvae being fed in 2013, hence fewer nymphs in 2014. Although we do not have 2013 DON data for our sites to directly test the hypothesis that tree masting prior to the start of our nymph sampling impacted the DON in the following years, we can propose that, as 2013 was a high mast year across northern England (MASTREE + ) [52], a similar chain of events could underlie the rise in DON we observed between 2014 and 2015 at sites like Chapel House.

Unlike DON, overall annual mean NIP values for 2014, 2015 and 2016 did not vary significantly from one another (Table S1), however, NIP did interact with site such that there were differences between years at Chapel House, Old Hall Wood but not at Linsty Green. This consistency is in keeping with some studies [42, 44, 45] but contrasts with others [25, 48, 53].

### Comparison of patterns at each site within study area

We observed a different abundance of nymphs (DON) at each site, with Linsty Green consistently supporting the highest number of nymphs and Chapel House consistently yielding the fewest nymphs. That tick abundance varies between survey sites is widely recognized, but far fewer studies have quantified to what extent these differing abundances are maintained year on year, i.e. do “ticky” sites remain “ticky” across different years? Our findings appear to align with the results of others, which indicate a degree of consistency, albeit with not-infrequent anomalies [e.g. 25, 31]. That our three sites, despite being close to one another, supported very different abundances of nymphs, emphasizes the importance of local determinants such as habitat structure and tick host community structure [45, 54]. The trend we observed matched that for local deer abundance, estimated by forestry rangers working on the sites, supporting the importance of deer in maintaining local tick populations [55].

The seasonal patterns of DON at different sites did vary. In particular, the average peak tick abundance at Chapel House was two months later than at Old Hall Wood and Linsty Green. This difference can be attributed to two factors. Firstly, delayed nymphal activity at Chapel House in 2016, with far higher nymphal abundances being recorded in August, September and October than in earlier months. Secondly, high levels of nymphal activity at Linsty Green and Old Hall Wood in early spring, particularly in 2015 and 2016. Why the seasonal dynamics of DON at Chapel House appeared different to those at Old Hall Wood and Linsty Green is unclear. However, Chapel House possessed markedly greater inter-annual variation in DON seasonal patterns than either of the other two sites, with the patterns for 2014 and 2015 being unimodal, peaking in June, whereas in 2016 the pattern was bimodal, with peaks in spring and late summer. As these 2014 and 2015 patterns were broadly akin to those observed at Old Hall Wood and Linsty Green, it appears that the anomaly at Chapel House is primarily the result of an atypical seasonal pattern in 2016. What provoked this anomaly and thus why the seasonal dynamics of DON at Chapel House appeared different to those as Old Hall Wood and Linsty Green is unclear. However, Old Hall Wood and Linsty Green are dense woodlands nestling on the valley floor whereas at Chapel House, tree cover is more sparce and the site lies on a high ridge with a south-westerly aspect and thus is likely more prone to DON determinants such as extremes of temperature and high saturation deficits [56]. Dramatic inter-annual variation in seasonal patterns of nymphal abundance at the same site, as we observed most clearly at Chapel House, has been repeatedly observed previously [31, 40] but concurrent quantification of such variation at different sites appears unstudied.

*B. burgdorferi* was present in ticks at all sites surveyed, but at a significantly different prevalence of infection between years within each site. For instance, Linsty Green consistently supported low prevalence of infection overall, and showed no significant differences in NIP between years whereas Chapel House consistently yielding the highest prevalence of infection, as has been frequently observed previously, with some variation in NIP between years [25, 31, 42]. The overall NIP at Chapel House was nearly 8%, three times greater than that at either of the other two sites. A possible explanation for this is that, as described above, whereas the sites at Old Hall Wood and Linsty Green lie well within established mixed woodland, the site at Chapel House is on the edge of a spruce woodland. Some studies have demonstrated higher NIP in tick populations inhabiting woodland-edge than in other habitats [e.g. 25] but others have found no correlation between NIP and habitat characteristics [e.g. 31].

We observed variations in the seasonal pattern of NIP at different sites, in particular an apparent spring peak at Chapel House that was absent from Old Hall Wood or Linsty Green. However, this anomaly may well be an artefact of small sample sizes in early spring surveys at Chapel House. Seasonal patterns at each site in different years did not appear to be very consistent with one another but, again, this may be the result of small sample sizes. However, significant year to year variation in NIP at some of our sites was observed, as has been reported previously; for example, Hartemink and colleagues [31] observed a greater than 10% change in annual NIP at 7/12 survey sites across The Netherlands between 2009 and 2016. What is perhaps particularly noteworthy is that two of the three sites in our study possessed “peak” years, when NIP was significantly higher than other years, but these were asynchronous; 2014 at Old Hall Wood, 2015 and 2016 (which were not significantly different from each other) at Chapel House, whereas there was a non-significant trend for higher NIP in 2016 at Linsty Green. It is this asynchrony that resulted in year-on-year variation in NIP not being apparent when data from all three sites were combined (see above). Similar idiosyncrasies have been recorded previously among sites close to one another in southern England, but small sample sizes precluded statistical support for the variation observed [25]. A larger dataset has been assembled for more disparate sites across the Netherlands [31] and examination of the summary of inter-site variation in annual NIP presented in this study also suggests asynchrony in NIP dynamics between sites. Thus, NIP appears to be determined by factors that vary on a very local (site-specific) scale, a conclusion that is not unsurprising given the importance of relative abundance of *B. burgdorferi* reservoir hosts such as small mammals or foraging birds on NIP. As this relative abundance is influenced not just by the population dynamics and competence of reservoir hosts but also by those of tick bloodmeal hosts that cannot sustain/transmit *B. burgdorferi*, it is inevitable that NIP will vary on a fine spatial scale (inter and probably intra-site).

Over the three years of our study, there was a non-significant trend for higher DIN at Linsty Green than in Old Hall Wood. As with DON and NIP, significant inter-site variation has been frequently reported previously, even for sites close to one another [25, 31]. The seasonal patterns of DIN at each site resemble one other more closely than the seasonal patterns for DON and NIP, primarily because the DON and NIP anomalies at individual sites, as discussed above, tended to balance each other out. Although both DON and NIP determine DIN, the year-on-year contributions in either of these metrics appeared to differ for each site. Previous studies, combining data from numerous study sites, have concluded that DON is the most important determinant of DIN [28, 30, 31] and although this may generally hold, it appears not to be consistent at the site level. For example, the marked rise in DIN at Linsty Green between 2015 and 2016 was to a greater extent a consequence of a significant rise in NIP, and a modest but non-significant rise in DON. On the other hand, the peak in DIN in Old Hall Wood in 2014 appears to be driven more by high NIP but low DON compared to other years. Why our observations differed from those in the studies cited above is unclear, although Tälleklint and Jaenson [30] suggested that the extent of correlation between DON and DIN may be dependent on local tick host community structure, particularly the relative abundance of deer. We acknowledge that our study did not differentiate between *B. burgdorferi* genospecies, which, due to their differing ecologies, are likely to exhibit different infection dynamics that may confound our observations and thus our interpretation of them.

## Conclusions

Our observations reinforce the complexity of tick and tick-borne pathogen ecology and emphasize how their dynamics can vary significantly even on a very local scale. Our understanding of the determinants of these dynamics remains very incomplete and thus predicting Lyme disease hazard continues to be challenging. Although some environmental (climate/habitat) characteristics have repeatedly been associated with Lyme disease hazard, these are, in part, proxies for the community structure of vertebrates that feed ticks and serve as reservoirs for *B. burgdorferi*. Direct, accurate quantification of this community structure is currently challenging, but such is its seminal value that exploration of new approaches to doing so should be pursued with vigour.

## Supplementary Information

Supplementary Material 1.

Supplementary Material 2.

## Data Availability

Data supporting the main conclusions of this study are included in the manuscript.
