# Supplementary material for: Asynchronous local differences in Lyme disease environmental hazard driven by variation in tick abundance and Borrelia burgdorferi infection prevalence
Source: Parasit Vectors. 2026 Jul 15;19:455. doi: 10.1186/s13071-026-07509-8 (PMC13681760; doi:10.1186/s13071-026-07509-8)
Supplement: Supplementary file 1 [file 13071_2026_7509_MOESM2_ESM.docx]

**Supplementary Information**

**Additional file 1: Fig S1:** relationship between observed (a) DON and DIN and (b) NIP and DIN, by site.

**Table S1:** Mean DON (SE), NIP (95% CI) and DIN (SE) for each site and year. Note that For Chapel House the following observations were excluded from the calculation: for DIN – Dec 2016 (unable to estimate prevalence in presence of positive DON); for NIP – Jan 2014, Feb 2014, Dec 2015, Jan 2015, Dec 2016 (each with less than 2 ticks tested).

|  |  | Year | | |
| --- | --- | --- | --- | --- |
| Site | metric | 2014 | 2015 | 2016 |
| Chapel House | DON | 8.91 (2.24) | 19.55 (6.36) | 25.64 (7.89) |
|  | NIP | 4.86 (3.28-6.9) | 9.23 (7.6-11.07) | 7.89 (6.27-9.78) |
|  | DIN | 0.44 (0.13) | 1.8 (0.64) | 2.02 (0.64) |
| Linsty Green | DON | 66.36 (10.02) | 66.91 (10.83) | 71.18 (14.8) |
|  | NIP | 2.02 (1.42-2.79) | 1.19 (0.75-1.8) | 2.72 (2.03-3.57) |
|  | DIN | 1.37 (0.38) | 0.71 (0.15) | 2.02 (0.62) |
| Old Hall Wood | DON | 19.27 (4.62) | 38.36 (7.03) | 27.82 (6.34) |
|  | NIP | 6.72 (5.17-8.56) | 1.41 (0.93-2.06) | 1.67 (1.08-2.45) |
|  | DIN | 1.19 (0.42) | 0.53 (0.2) | 0.43 (0.08) |
